# “Complete Venous Shutdown:” A Rare Case of Combined Superior Vena Cava (SVC) and Inferior Vena Cava (IVC) Occlusion

**DOI:** 10.1155/2023/5590280

**Published:** 2023-12-15

**Authors:** M. Kasim Malik, Wajahat Humayun, Amir Darki

**Affiliations:** ^1^Department of Internal Medicine, Loyola University Medical Center, 2160 S 1st Avenue, Maywood, IL 60153, USA; ^2^Division of Cardiology, Department of Internal Medicine, Loyola University Medical Center, 2160 S 1st Avenue, Maywood, IL 60153, USA

## Abstract

Independently, superior vena cava (SVC) occlusion and inferior vena cava (IVC) occlusion are usually seen in the setting of SVC syndrome and iliocaval venous obstruction (ICVO), respectively. Concomitant occlusion of the SVC and IVC is rare and most commonly seen in the setting of malignancy or other hypercoagulable states. Venous hypertension can lead to the formation of “downhill” varices in the esophagus and can be a rare source of gastrointestinal bleeding. We present a rare case of combined SVC and IVC occlusion and its management.

## 1. Introduction

Concomitant superior vena cava (SVC) and inferior vena cava (IVC) occlusion most commonly occurs in the setting of malignancy but can also be seen in other hypercoagulable states or can be iatrogenic in the setting of central venous catheterization [1]. Depending on the site of occlusion, clot burden, and chronicity, SVC and IVC occlusion can present with a spectrum of symptoms. Most commonly, SVC occlusion presents with SVC syndrome manifesting with upper extremity and/or facial edema, plethora, and vascular distension ultimately progressing to functional impairment, cerebral edema, airway compromise, and shock if untreated [2]. IVC occlusion is less common and more frequently seen in iliocaval venous obstruction (ICVO) in the setting of May-Thurner syndrome, foreign body (IVC filter), malignancy, or thrombosis. Like SVC occlusion, the clinical presentation of ICVO depends on the clot burden and chronicity of occlusion with typical symptoms being lower extremity edema, pain, and venous insufficiency [3, 4]. Management of SVC and ICVO includes angioplasty, stenting, thrombolytics, and vascular bypass [1, 4]. Combined SVC and IVC obstruction is extremely rare with only case report data available, and thus, there is extremely limited information regarding its management.

## 2. Case Presentation

Our patient is a 35-year-old male with a medical history significant for autosomal dominant adult polycystic kidney disease status postkidney transplant complicated by graft failure now on hemodialysis (HD) requiring recurrent central venous catheters in the upper and lower extremities, SVC occlusion, heart failure with preserved ejection fraction, proximal lower extremity deep venous thrombosis (DVT), and subacute pulmonary embolism who was transferred to our institution for further evaluation.

The patient had presented to an outside hospital with septic shock and was found to have a proteus mirabilis central line-associated bacterial infection from his right subclavian HD catheter. He also had an upper gastrointestinal bleed (UGIB) treated with esophagogastroduodenoscopy (EGD) that was notable for esophageal varices and acute hypoxic respiratory failure secondary to acute pulmonary edema.

Upon arrival at our institution, the patient had stable vitals: temperature 97.7°F, BP 127/92 mmHg, HR 72 bpm, and O_2_ saturation of 94% on room air. An infectious disease workup demonstrated negative surveillance blood cultures; therefore, a new HD catheter was placed. Due to his history of DVT, a Doppler ultrasound of the lower extremities was performed and notable for chronic DVT of the right distal external iliac, common femoral, femoral, and popliteal veins.

In the following day, the patient had an episode of hemoptysis complicated by hemorrhagic shock while having a computed tomography (CT) venogram which required emergent intubation. An emergent EGD was complicated by cardiac arrest (pulseless electrical activity) for which a successful return of spontaneous circulation was obtained. The EGD was notable for downhill esophageal varices secondary to known SVC occlusion, and a massive transfusion protocol was initiated. Interventional radiology (IR) was consulted for consideration of emergent transjugular intrahepatic portosystemic shunt (TIPS); however, the patient was not felt to be a candidate given his extensive central venous stenosis. An urgent hepatology evaluation demonstrated no evidence of liver disease or portal hypertension.

**Figure 1:**
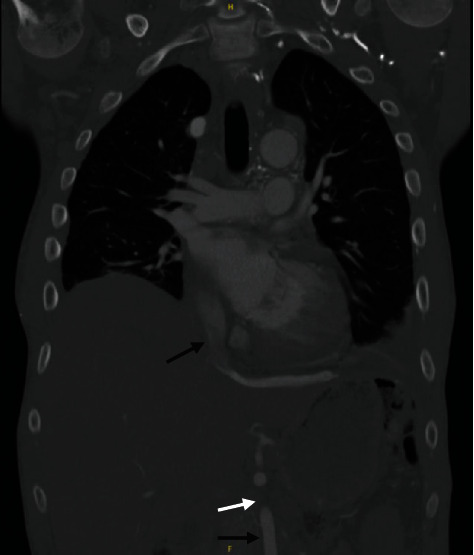
CT chest/abdomen/pelvis. CT scan demonstrating stenosis of a collateral to the IVC (white arrow). The IVC is indicated by the top black arrow. A collateral to the IVC is indicated by the bottom black arrow.

**Figure 2:**
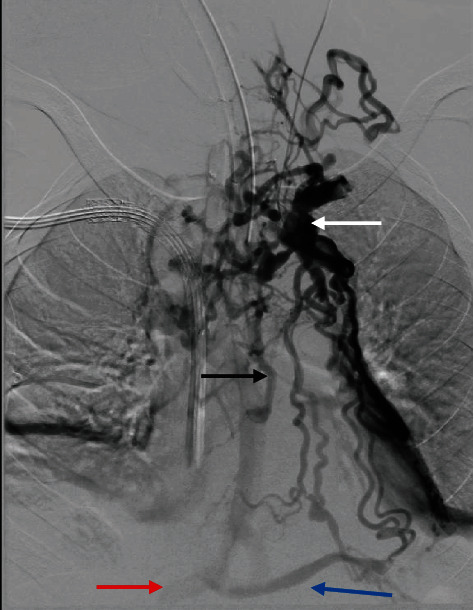
Interventional radiology venogram. Venogram demonstrating occlusion of the central portion of the left brachiocephalic vein with collateral flow from the peripheral remnant of the left brachiocephalic vein (white arrow) into a prominent collateral of the hemiazygos vein (black arrow) bypassing the occluded superior vena cava. Collateral flow from the left brachiocephalic vein can also be seen entering the left lower posterior intercostal vein (blue arrow). The inferior vena cava is indicated by the red arrow.

A CT of the chest/abdomen/pelvis was performed and was notable for narrowing of the IVC superior to the bifurcation. Additionally, dilated esophageal varices were seen in the left hemiabdomen which communicated with the IVC at the level of the superior mesenteric artery (Figure 1). IR performed a venogram demonstrating central occlusion with collateralization and thrombus present with no communication between the left brachiocephalic vein and SVC (Figure 2). Left brachiocephalic vein/SVC recanalization was attempted and failed by IR. Hematology was consulted, and a hypercoagulability workup was performed which came back negative. His hospital course was further complicated by recurrent UGIB requiring a total of four EGDs. Cardiovascular surgery was consulted, and he underwent azygous to right atrium bypass to decompress the central venous hypertension. A chronological sequence of diagnostic imaging and interventional procedures is listed in Table 1. He made a complete recovery and was eventually discharged on postoperative day thirty after a total of forty-five days in the hospital.

## 3. Discussion

Independently, SVC occlusion is usually seen in the setting of SVC syndrome, and IVC occlusion is most commonly seen in the setting of ICVO. This case demonstrates a rare presentation of combined SVC and IVC occlusion that was incidentally found on CT imaging and venography with collateralization through a prominent azygous system.

Very limited data on the incidence of combined SVC and IVC occlusion have been published. There are few case reports that have been published documenting that this has occurred in patients with known hypercoagulable states including prothrombin gene mutation G20210A and APLS [5, 6].

This case also highlights the importance of evaluation for central venous hypertension in the setting of esophageal varices when there is no evidence of portal hypertension or liver cirrhosis. Portal hypertension is the most common cause of esophageal varices, referred to as “uphill” varices, and is usually found in the lower third of the esophagus, contrary to “downhill” varices which are seen in the upper third of the esophagus in the setting of SVC obstruction [7]. In the setting of SVC obstruction, the backup of pressure into the upper esophagus leads to varices and is a potentially rare cause of UGIB.

Given the rarity of this presentation, there is limited published data regarding management. This patient failed endovascular SVC recanalization and required surgical bypass from the azygous to the right atrium and had a complete recovery.

## 4. Conclusion

Combined SVC and IVC occlusion is a rare phenomenon most commonly seen in malignancy and cases of thrombophilia. This case presentation adds to the limited published data regarding this clinical scenario and its management. We present a novel surgical bypass procedure adding to the limited published data regarding combined SVC and IVC occlusion management. Future directions include gathering case reports and creating a clinical case series in order to help create proposed management algorithm.

## Figures and Tables

**Table 1:** Sequence of diagnostic and interventional modalities.

Imaging/intervention	Results/outcomes
1. Initial outside hospital EGD	Esophageal varices
2. Doppler ultrasound of lower extremities	Chronic DVTs in the right lower extremity
3. Second EGD (after hemoptysis)	Downhill varices identified
4. CT chest/abdomen/pelvis	IVC stenosis identified; dilated esophageal varices with communication to the IV
5. IR venogram	Central occlusion and thrombus of SVC with collateralization
6. Surgical bypass	Successful central venous decompression

## Data Availability

The data used to support the findings of this study are included within the article.
